# The development of nonalcoholic steatohepatitis is subjected to breeder dependent variation in guinea pigs

**DOI:** 10.1038/s41598-021-82643-0

**Published:** 2021-02-03

**Authors:** D. H. Ipsen, R. H. Agerskov, J. H. Klaebel, J. Lykkesfeldt, Pernille Tveden-Nyborg

**Affiliations:** Department of Veterinary and Animal Sciences, Faculty of Health and Medical Sciences, University of Copenhagen, Ridebanevej 9, 1870 Frederiksberg C, Denmark

**Keywords:** Experimental models of disease, Non-alcoholic fatty liver disease, Non-alcoholic steatohepatitis

## Abstract

Variability in disease development due to differences in strains and breeders constitutes a substantial challenge in preclinical research. However, the impact of the breeder on non-alcoholic steatohepatitis (NASH) is not yet fully elucidated. This retrospective study investigates NASH development in guinea pigs from Charles River or Envigo fed a high fat diet (20% fat, 15% sucrose, 0.35% cholesterol) for 16 or 24/25 weeks. Charles River animals displayed more severe NASH, with higher steatosis (p < 0.05 at week 16), inflammation (p < 0.05 at both week), fibrosis (p < 0.05 at week 16) and disease activity (p < 0.05 at both weeks). Accordingly, alanine and aspartate aminotransferase were increased at week 24/25 (p < 0.01). Hepatic expression of inflammatory (*Ccl2*, *Cxcl8*) and fibrotic (*Pdgf*, *Serpine1*, *Col1a1*) genes was also increased (p < 0.05). Differences were observed in healthy chow (4% fat, 0% sucrose, 0% cholesterol) fed animals: Envigo animals displayed higher relative liver weights (p < 0.01 at both weeks), liver cholesterol (p < 0.0001 at week 24/25) and aspartate aminotransferase (p < 0.05 at week 16), but lower levels of alkaline phosphatase (p < 0.0001 at week 24/25). These findings accentuates the importance of the breeder and its effect on NASH development and severity. Consequently, this may affect reproducibility, study comparison and limit the potential of developing novel therapies.

## Introduction

Susceptibility to disease development and progression is highly individual both in humans and in animal models. While this variability is a natural part of biology, it constitutes a substantial challenge when conducting preclinical research increasing the necessary sample size and complicating comparison of studies and their reproducibility. Non-alcoholic steatohepatitis (NASH) is one of the most frequent chronic liver diseases^1^. It can progress to liver fibrosis with deteriorating liver function and has been projected to become the leading cause of hepatocellular carcinoma in the United States^2^. Disease development is remarkably heterogeneous with patients being characterized as rapid or slow progressors depending on how fast they develop severe liver fibrosis^3^. Several studies in animal models have highlighted that the breeder and the strain may influence insulin sensitivity as well as the susceptibility to develop hepatic steatosis, inflammation and fibrosis^4–6^. Genetic makeup does not account for all the disparities between strains since disease development in ‘identical’ strains also varies significantly between breeders, suggesting a pronounced breeder-effect on model phenotype. Likewise, different breeders supply mice with different airway responsiveness, gut microbiota and susceptibility to malaria or rats with dissimilar infarct volume caused by focal ischemia^7–9^. Even animals from the same breeder do not necessarily develop a similar phenotype e.g. neurotransmitter levels and seizure susceptibility differed between rats from various breeding locations of the same vendor^10^.

In line with the rapid increase in NASH patients and the current lack of treatment targeting NASH-induced liver fibrosis, studies in preclinical disease models have intensified. The guinea pig is an excellent model of diet-induced NASH, distinguishing itself by developing advanced hepatic fibrosis within a relatively short timeframe^11–16^. While the effects of strain is recognized to affect NASH development in other rodent species, relatively little is known regarding the impact of the breeder and its influence on the development of NASH and liver fibrosis. Reviewing data sampled from independent studies performed at the same laboratory facility, this retrospective study is the first to investigate if NASH development and progression differs in guinea pigs obtained from two different commercial breeders.

## Results

### Body weight

**Figure 1:**
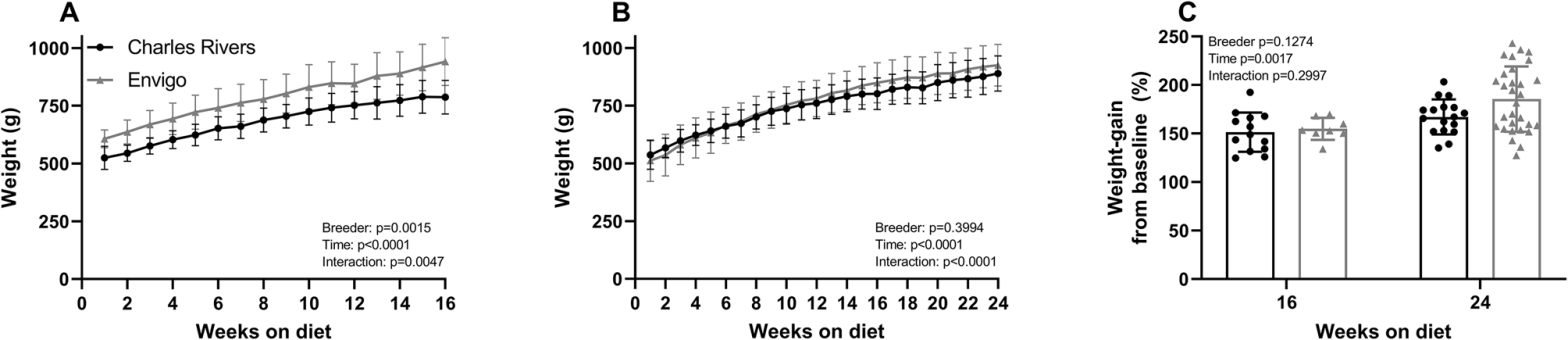
Body weight and relative weight-gain. (**A**) Bodyweights were higher for guinea pigs fed a high fat diet from Envigo compared with Charles River from week 1 to 16. (**B**) Bodyweights did not differ between guinea pigs fed a high fat diet for 1–24 weeks. (**C**) Weight-gain relative to baseline (to account for dissimilar starting weights) eliminated the difference between breeders at week 16. Analysed by mixed effect model (**A**,**B**) or 2-way ANOVA (**C**).

Body weights were higher in animals from Envigo upon arrival compared with Charles River fed a high fat diet for 16 weeks due to Envigo animals being delivered at a slightly higher weight-range than animals delivered from Charles River (Fig. 1A). The body weight of animals fed a high fat diet for 24 weeks did not differ between the two breeders (Fig. 1B). Following normalization to baseline, Envigo and Charles River animals did not differ in relative weight (data not shown) or relative weight gain (Fig. 1C). Individual values from each study are shown in Supplemental Figure S1.

### Liver histology and status

Intra-observer scoring of NASH animals did not change over time, as there was good agreement between the initial grade and the re-grading for all parameters: steatosis κ = 0.89, ballooning κ = 0.82, lobular inflammation κ = 0.71, portal inflammation κ = 0.61 and fibrosis κ = 0.92.

**Figure 2:**
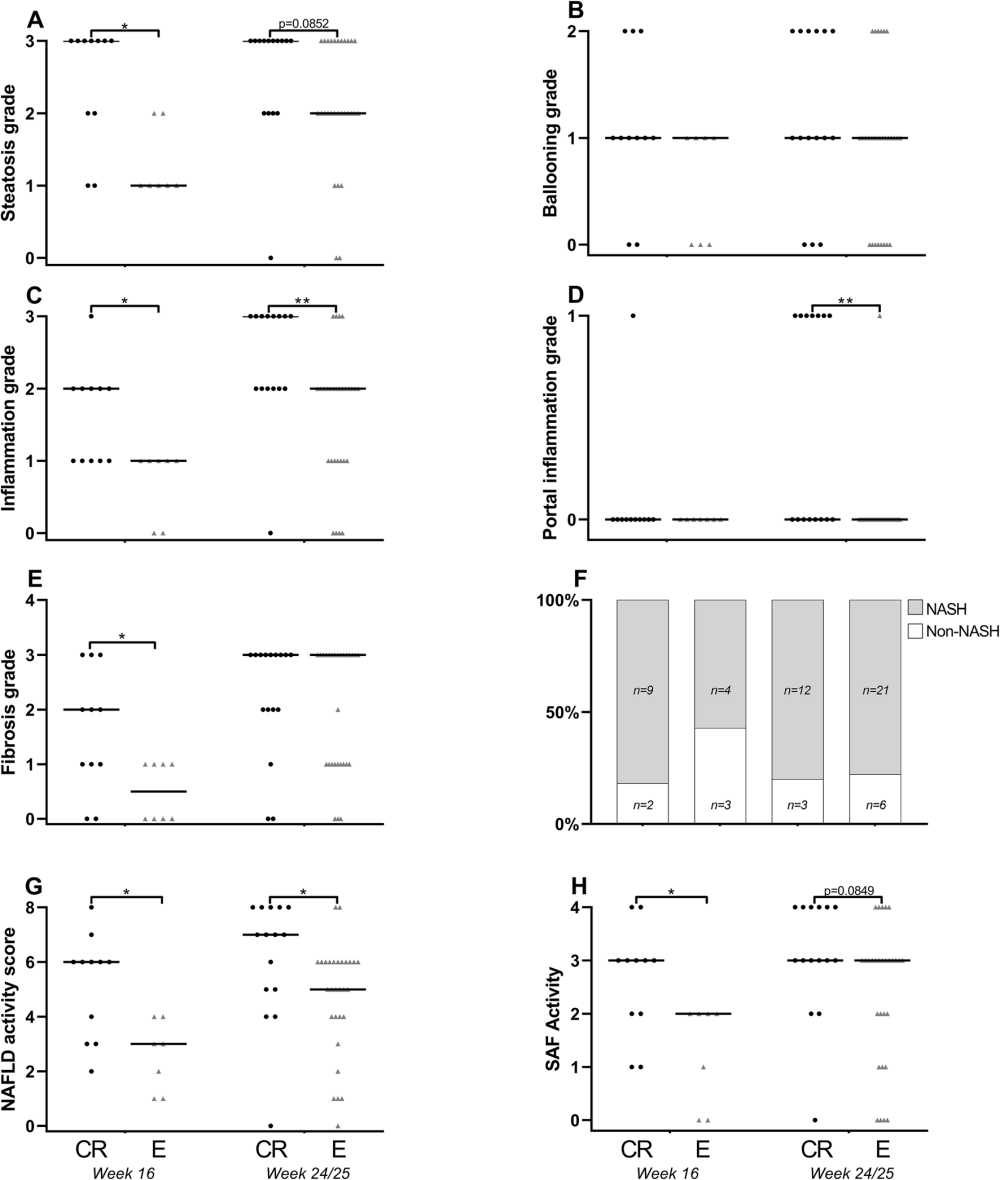
Liver histology and disease severity. Guinea pigs from Charles River displayed an overall more severe NASH phenotype compared with animals from Envigo. (**A**) Steatosis was higher in guinea pigs from Charles River at week 16, (**B**) while hepatocyte ballooning did not differ between animals from the two breeders. (**C**) Lobular inflammation was enhanced at both time-points in animals from Charles River and (**D**) portal inflammation was increased at week 25 compared with Envigo. (**E**) At week 16, fibrosis grade was higher in Charles River, but at week 25 it was similar to Envigo. (**F**) There was no difference between breeders in the amount of animals that developed NASH (defined as the simultaneous presence of steatosis, ballooning and lobular inflammation) at any time-point. (**G**,**H**) In accordance with the liver histology, animals from Charles River displayed higher disease activity at week 16 and 24/25 when calculated as the NAS and at week 16 when calculated as the SAF activity. Line represents medians. *p < 0.05 **p < 0.01 compared between breeders at the same time-point. Histological scores were analysed at each time-point by Mann–Whitney test (**A**–**E**,**G**,**H**) and frequencies (**F**) by Fishers exact test. *CR *Charles River*,. E *Envigo.

**Figure 3:**
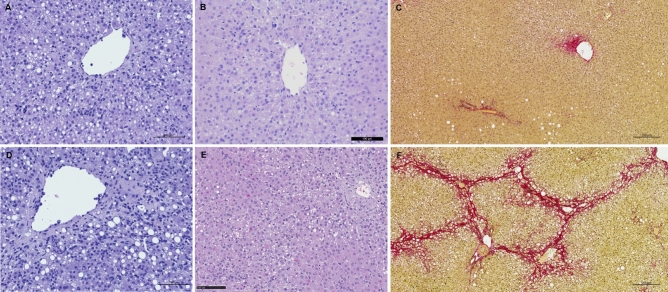
Liver histology. Representative images of Charles River and Envigo animals with NAS similar to group medians at week 16 and 24/25. (**A**) Charles River animal with NAS = 6 at week 16. (**B**) Envigo animal with NAS = 3 at week 16. (**C**) Charles River animal with NAS = 7 at week 24/25. (**D**) Envigo animal with NAS = 5 at week 24/25. Representative images of mild F1 (**E**) and bridging (advanced) F3 (**F**) fibrosis. Scale bar is 100 μm (**A**,**B**,**D**,**E**) or 200 μm (**C**,**E**). *NAS *non-alcoholic fatty liver disease activity score.

Guinea pigs from Charles River had a significantly higher steatosis grade (median grade 3) after 16 weeks compared with Envigo (median grade 1) (p = 0.0128). A similar tendency was present after 24/25 weeks, but did not reach statistical significance (p = 0.0852) (Fig. 2A). Hepatocellular ballooning did not differ at any time point between animals from the two breeders (Fig. 2B). Animals from Charles River had increased lobular inflammation compared with Envigo at both week 16 (median grade 2 vs. median grade 1, p = 0.0154) and week 24/25 (median grade 3 vs. median grade 2, p = 0.0040) (Fig. 2C). At week 24/25, portal inflammation was also significantly higher in animals from Charles River compared with Envigo (p = 0.0011) (Fig. 2D). Fibrosis grade was higher in guinea pigs from Charles River (median grade 2) compared with Envigo (median grade 0.5) after 16 weeks (p = 0.0301), but was not different after 24/25 weeks (p = 0.5854) (Figs. 2E, 3). The number of animals with severe (F ≥ 3) or none-to-moderate (F ≤ 2) fibrosis did not differ between the two breeders at any time-point (data not shown). The number of animals diagnosed with NASH or non-NASH was not different at week 16 (p = 0.3260) or 24/25 (p > 0.9999) (Fig. 2F). Disease activity calculated as NAS was significantly increased in guinea pigs from Charles River at both week 16 (p = 0.0119) and 25 (p = 0.0164) (Figs. 2G, 3). Excluding the steatosis component and calculating the disease activity based on the SAF produced similar results, with higher disease activity in guinea pigs from Charles River at week 16 (p = 0.0132) and nominally higher activity at week 24/25 (p = 0.0849) compared with Envigo animals (Fig. 2H). The differences in NASH-related liver histology between Charles River and Envigo was not driven by any single study with extreme values (Supplemental Figure S2). Liver histology was not statistically different between healthy chow fed animals from the two breeders: Chow fed control animals from Charles River and Envigo did not have steatosis (a single Envigo animal had grade 1 steatosis at week 16) or hepatocyte ballooning at either time point. For both breeders, median inflammation grade was 1 and 0.5 at week 16 and 24/25, respectively. None of the control animals exhibited fibrosis, except a single animal with F1 from Charles River at week 25 (data not shown).

### Liver status and dyslipidaemia

**Figure 4:**
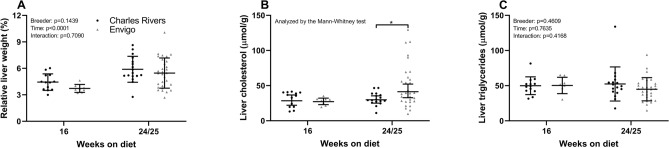
Liver weight and lipid content. (**A**) Liver weight relative to bodyweight did not differ between Charles River and Envigo animals. (**B**) Envigo animals displayed a higher hepatic cholesterol content compared with Charles River at week 25, (**C**) while liver triglyceride levels were not different between breeders. Means with standard deviations. *p < 0.05 compared between breeders at the same time-point. Analysed by 2-way ANOVA (**A**,**C**) or Mann–Whitney test (**B**) as variance homogeneity could not be obtained following transformation.

Liver weight relative to body weight and liver triglyceride content did not differ between breeders while liver cholesterol levels were higher in Envigo animals at week 24/25 (p = 0.0474) (Fig. 4). This difference in liver cholesterol may be attributable to a single study (Envigo 2019, see “Methods”), in which animals had higher hepatic cholesterol levels compared to similar studies utilizing animals from Envigo (Envigo 2017 and Envigo 2017/2018, see “Methods”) (Supplemental Figure S3). Circulating alanine aminotransferase (ALT) levels were higher in guinea pigs from Charles River after 24/25 weeks (p < 0.0001) (Table 1). Overall aspartate aminotransferase (AST) levels were higher in Charles River animals (p_breeder_ = 0.0137) and post-hoc comparison revealed higher levels at week 24/25 in animals from Charles River (p = 0.0028). Overall plasma cholesterol was higher in animals from Charles River (p_breeder_ = 0.0360), but post-hoc testing did not reveal a significant difference at any specific time-point. Plasma ALP, triglycerides and free fatty acids did not differ between the two breeders.

**Table 1:** Liver enzymes and plasma lipids in high fat fed guinea pigs with NASH.

	Week	Charles River	Envigo	2-way ANOVA
ALT (U/l)	16	93.53 (76.30–114.7)	96.43 (83.53–111.3)	Breeder: p = 0.0095 Time: p = 0.1182 Interaction: p = 0.0037
	24/25	106.5 (86.04–131.9)	63.47 (56.98–70.70)****	
AST (U/l)	16	381.5 (282.8–514.7)	299.3 (239.6–373.9)	Breeder: p = 0.0137 Time: p = 0.0527 Interaction: p = 0.2761
	24/25	640.5 (467.1–878.4)	347.0 (268.6–448.2)**	
ALP (U/l)	16	52.85 ± 11.10	56.33 ± 20.50	Breeder: p = 0.3483 Time: p < 0.0001 Interaction: p = 0.0450
	24/25	41.71 ± 8.39	32.24 ± 6.43	
Plasma cholesterol (μM)	16	7.71 ± 2.24	6.12 ± 2.21	Breeder: p = 0.0360 Time: p = 0.0096 Interaction: p = 0.6773
	24/25	5.79 ± 2.19	4.72 ± 2.24	
Plasma triglyceride (μM)	16	0.67 ± 0.20	0.78 ± 0.31	Breeder: p = 0.0705 Time: p = 0.0083 Interaction: p = 0.9031
	24/25	0.51 ± 0.11	0.61 ± 0.24	
Plasma free fatty acids (μM)	16	0.56 ± 0.13	0.64 ± 0.14	Breeder: p = 0.2481 Time: p = 0.9767 Interaction: p = 0.5529
	24/25	0.59 ± 0.14	0.61 ± 0.18	

Means with standard deviations or geometic means with 95% confidence intervals. n_Charles River week 16_ = 13, n_Envigo week 16_ = 7–8, n_Charles River week 24/25_ = 17, n_Envigo week 24/25_^=^29–30. ALP at week 16 n = 3 for Envigo only.

*ALP* alkaline phosphatase*, ALT* alanine aminotransferase, *AST* aspartate aminotransferase.

**p < 0.01 ****p < 0.0001 vs Charles River at same time point. Analysed by two-way ANOVA with Sidak’s multiple comparison test.

Values from parallel chow fed controls are shown in Table 2. Compared with Envigo, chow fed Charles River animals had lower relative liver weight at week 16 (p = 0.0032) and 24/25 (p = 0.0013) and hepatic cholesterol content was lower at week 24/25 (p < 0.0001). Hepatic triglyceride levels did not differ between breeders. Plasma levels of ALT, triglycerides and free fatty acids did not differ between healthy control animals from Charles River and Envigo. Overall AST levels were significantly lower in chow fed guinea pigs from Charles River (p_breeder_ = 0.0046) and post-hoc testing revealed that, specifically, levels were lower at week 16 (p = 0.0169). Overall ALP levels were higher in Charles River animals (p_breeder_ < 0.0001) with post-hoc comparison showing a significant difference at week 24/25 (p < 0.0001). Plasma cholesterol was higher in chow fed animals from Charles River at week 16 (p = 0.0004).

**Table 2:** Liver status and plasma lipids in healthy chow fed guinea pigs.

	Week	Charles River	Envigo	2-way ANOVA
Relative liver weight (%)	16 24/25	2.17 ± 0.11 2.26 ± 0.28	2.61 ± 0.32** 2.69 ± 0.19**	Breeder: p < 0.0001 Time: p = 0.2997 Interaction: p = 0.9361
Liver cholesterol (μmol/g tissue)	16 24/25	6.90 ± 0.53 5.40 ± 1.11	7.31 ± 0.59 7.97 ± 0.80****	Breeder: p < 0.0001 Time: p = 0.2348 Interaction: p = 0.0016
Liver triglyceride (μmol/g tissue)	16 24/25	39.16 ± 26.19 17.07 ± 11.01	20.54 ± 6.31 27.46 ± 25.42	Breeder: p = 0.5171 Time: p = 0.2357 Interaction: p = 0.0276
ALT (U/l)	16	43.16 (35.01–53.21)	59.25 (41.45–84.69)	Breeder: p = 0.1758 Time: p = 0.2015 Interaction: p = 0.3016
	24/25	41.76 (30.90–56.43)	43.62 (36.21–52.54)	
AST (U/l)	16	67.09 (40.93–110.0)	222.0 (107.5–458.4)*	Breeder: p = 0.0046 Time: p = 0.4476 Interaction: p = 0.2549
	24/25	74.95 (38.88–144.5)	128.4 (80.08–205.9)	
ALP (U/l)	16	70.29 ± 5.53	64.80 ± 5.26	Breeder: p < 0.0001 Time: p < 0.0001 Interaction: p = 0.0029
	24/25	55.58 ± 6.82	36.00 ± 4.50****	
Plasma cholesterol (μM)	16	1.34 ± 0.30	0.83 ± 0.14***	Breeder: p = 0.0064 Time: p = 0.0483 Interaction: p = 0.0017
	24/25	0.90 ± 0.28	0.94 ± 0.15	
Plasma triglycerides (μM)	16	2.51 ± 1.96	1.93 ± 0.34	Breeder: p = 0.5910 Time: p = 0.0239 Interaction: p = 0.2290
	24/25	1.32 ± 0.42	1.55 ± 0.59	
Plasma free fatty acids (μM)	16	0.77 ± 0.23	0.52 ± 0.16	Breeder: p = 0.4845 Time: p = 0.2697 Interaction: p = 0.0070
	24/25	0.49 ± 0.17	0.64 ± 0.24	

Means with standard deviations or geometic means with 95% confidence intervals. n_Charles River week 16_ = 7, n_Envigo week 16_ = 8, n_Charles River week 24/25_ = 12, n_Envigo week 24/25_ = 8. ALP at week 16 n = 5 for Envigo only.

*ALP* alkaline phosphatase, *ALT* alanine aminotransferase, *AST* aspartate aminotransferase.

*p < 0.05, **p < 0.01 ***p < 0.001 ****p < 0.0001 vs Charles River at same time point. Analysed by two-way ANOVA with Sidak’s multiple comparison test.

To ensure that differences in disease development were not merely a reflection of different baseline values—i.e. the healthy control animals were not similar between breeders—continuous data from high fat fed animals were normalized to control animals from the same breeder at the same time-point (data not shown). Normalized data accentuated the already established differences and similarities in plasma ALT, AST, total cholesterol and free fatty acids, with normalized ALT and AST levels also being higher in Charles River animals at week 16 (p = 0.0458 and p < 0.0001, respectively) (data not shown). Following normalization, hepatic cholesterol content and plasma triglycerides no longer differed between breeders. In contrast, normalized relative liver weights were higher in animals from Charles River compared with Envigo at week 16 (p = 0.0118) and 24/25 (p = 0.0042). Normalized hepatic triglycerides were lower at week 16 (p = 0.0002) and higher at week 25 (p < 0.0001) in guinea pigs from Charles River compared to Envigo. Normalized ALP at week 25 were lower in guinea pigs from Charles River compared with Envigo (p = 0.0166).

### Hepatic gene expression

**Figure 5:**
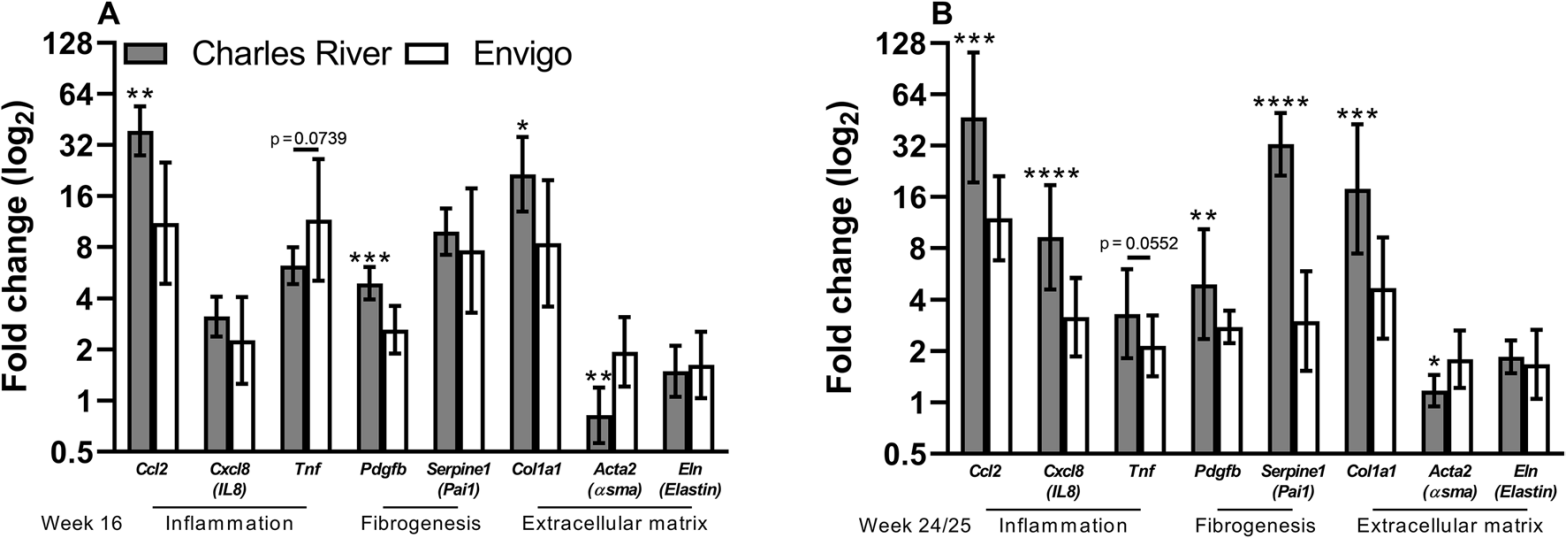
Hepatic gene expression in animals with NASH. Gene expression in liver samples from animals with NASH from Charles River or Envigo relative to healthy to control from the respective breeders. (**A**) At week 16, most genes were statistically significant or normally increased in animals from Charles River compared Envigo. (**B**) At week 24/25, this effect was even more pronounced with animals from Charles River displaying clear increases in genes related to inflammation, fibrogenesis and extracellular matrix composition. Mean fold changes with ranges. The scale of the y-axis is log_2_. Analysed by unpaired t-test or Welch’s t-test in case of heterogeneous variance. *p < 0.05 **p < 0.01 ***p < 0.001, ****p < 0.0001 compared to Envigo animals. *αsma* α smooth muscle actin, *Acta2* actin alpha 2, *Ccl2* Chemokine (C-C motif) ligand 2, *Col1a1* Collagen 1a1, *Cxcl8* Chemokine (C-X-C motif) ligand 8, *Eln* Elastin, *IL8* Interleukin 8, *Pai11* Plasminogen activator inhibitor 1, *Pdgfb* Platelet-derived growth factor b, *Serpine1* Serpin Family E Member 1, *Tnf * Tumor necrosis factor.

At week 16 *Ccl2* [also known as monocyte chemoattractant protein 1 (*Mcp1*)], *Pdgfb* and *Col1a1* were increased approximately 3.5-, 2- and 2.5-fold in Charles River animals with NASH compared to Envigo, respectively (p = 0.0030, p = 0.0008 and p = 0.0253, respectively) (Fig. 5A). *Acta2* (α smooth muscle actin) was increased in NASH animals from Envigo compared to Charles River (p = 0.0020). At week 24/25, the inflammatory genes *Ccl2* and *Cxcl8* (interleukin 8) were increased approximately 4- and 3-fold in Charles Rivers NASH animals (p = 0.0002 and p = 0.004) while *Tnf* was nominally increased compared to Envigo animals with NASH (p = 0.0552) (Fig. 5B). The fibrogenic genes *Pdgfb* and *Serpine1* [plasminogen activator inhibitor 1 (*Pai1*)] and the extracellular matrix-related gene *Col1a1* were all upregulated in the livers of Charles River animals with NASH compared to Envigo animals (p = 0.0071, p < 0.0001 and p = 0.0003, respectively). Again, *Acta2* was increased in animals from Envigo compared to Charles River (p = 0.0196). *Eln* expression did not differ between animals with NASH from the two breeders at either time point.

## Discussion

This retrospective study finds considerable differences in liver histology, hepatic expression of inflammatory and fibrogenic genes, liver enzymes and circulating lipids in guinea pigs from two commercial breeders although exposed to the same dietary regime and housing conditions. Guinea pigs from Charles River and Envigo both developed NASH, however, the phenotype was more severe in animals from Charles River, which developed more hepatic steatosis, inflammation and fibrosis. Accordingly, hepatic expression of genes related to inflammation and fibrosis were also higher in Charles River animals. In addition, levels of ALT, AST and circulating cholesterol was increased in guinea pigs from Charles River compared to Envigo. These findings reiterate the influence of breeders on experimental outcomes in NASH models and may help explain the challenges in reproducing results even when using seemingly ‘identical’ animals.

Breeder source clearly affected the severity of NASH and dyslipidaemia in guinea pigs. This is in line with previous studies finding variation in several disease parameters such as malaria susceptibility, faecal microbiota, depression development, behavioural tests as well as neuropathic pain phenotype and drug response in mice or rats sourced from different vendors^8,17–20^. Collectively, this emphasizes that the origin of the experimental animal is a significant contributor to experimental outcome. The diagnosis of NASH is based exclusively on liver histology, which also serves as the main endpoint in clinical trials and makes it a key focus in preclinical models as well. Guinea pigs from Charles River developed more severe hepatic steatosis, inflammation, fibrosis and disease activity expressed both as NAS and SAF activity compared with guinea pigs from Envigo. In accordance with the more severe hepatic inflammation, expression of *Ccl2*—a key regulator of monocyte/macrophage recruitment to the liver- was more increased in the livers of animals with NASH from Charles River than animals from Envigo. Similarly, and in agreement with a higher fibrosis grade, *Pdgfb* and *Cola1a* expression was higher in Charles River animals. These differences became even more apparent at week 24/25. Hepatic inflammation progressed in animals from both breeders, but inflammation grade, *Ccl2*, *Cxcl8* (interleukin 8) and *Tnf* levels were higher in Charles Rivers animals, corroborating the more severe phenotype. While the median fibrosis grade was the same across breeders at week 24/25, pro-fibrogenic [*Pdgfb* and *Serpine1* (PAI1)] and extracellular matrix-related (*Col1a1*) genes were increased to a higher degree in animals from Charles River compared to animals from Envigo. Thus, differences in histopathology may be explained, at least partially, by a stronger recruitment of inflammatory cells to the liver (mediated by *Ccl2* and *Cxcl8*) and more potent fibrogenic signalling (mediated by *Pdgfb and Serpine1)*. However, the underlying causes for this augmented signalling remains to be further investigated. In contrast to the more severe expression of other fibrogenic genes, α smooth muscle actin (*Acta2*)—a marker of activated hepatic stellate cells—was lower in Charles River compared to Envigo animals. Recently, α smooth muscle actin was reported to only target a subpopulation of activated hepatic stellate cells and it could be speculated that this specific subpopulation contributed more to fibrosis in Envigo animals^21^. Further substantiating the more serious phenotype and in agreement with the differences in liver histology, levels of ALT and AST were also higher in guinea pigs from Charles River. Notably, histological and biochemical endpoints can differ between the lobuli of the liver and even within the same lobule in both mice and guinea pig NASH models^22^. However, liver samples for histology and biochemical analyses were collected from within 0.5–1 cm of the same location in the left lateral lobe, excluding the possibility that the observed breeder differences are caused by sampling variability.

The frequency of NASH-diagnosis was similar in guinea pigs from both breeders, although nominally more Charles River guinea pigs developed NASH at week 16. Disease severity varied in animals from both breeders and is in line with the substantial inter-patient variation in disease development and fibrosis progression recorded in humans^3^, suggesting that animals from both breeders mimic the natural progression and variability of NASH patients. Similarly, high fat fed C57BL/6 mice, from the same breeder, separated into low-responders with moderate steatosis and high-responders characterized by pronounced NASH with increased hepatic expression of inflammatory and fibrogenic genes^23^. High-responders also displayed adipose tissue dysfunction and an early increase in circulating leptin indicating that NASH susceptibility and adipose tissue dysfunction is closely related^23^. In addition to this, the gut microbiome is altered in patients with NASH and has been suggested to contribute to disease progression^24^. However, the breeder imposes a significant effect on the composition of both the bacterial and viral gut community^25^. Differences in the gut microbiota may account for some of the differences observed in the present study between animals from Charles River and Envigo. Thus, measures of gut microbiome alterations and adipose dysfunction deserve further investigation in future studies. Although genetic composition is a likely contributor, the mechanisms explaining the reported substantial breeder heterogeneity in diet-induced NASH remains to be elucidated. In mice, inter-strain susceptibility to NASH was linked to hepatic methylation phenotype suggesting that epigenetic status determine NASH development^26^. Compared with patients with no to moderate fibrosis, anti-fibrotic genes (peroxisome-proliferator activated receptor α and -δ) were hypermethylated, while pro-fibrotic genes (transforming growth factor β, collagen 1A1 and platelet-derived growth factor α) were hypomethylated in patients with progressive fibrosis indicating lower and higher expression, respectively^27^. Interestingly, expression of *Col1a1* and *Pdgfb* was also increased in guinea pigs with severe NASH from Charles River compared to animals with less severe NASH from Envigo. Furthermore, several genetic variants have been associated with NAFLD development and progression, especially patatin-like phospholipase domain-containing protein 3 (PNPLA3), transmembrane 6 superfamily member 2 (TM6SF2), membrane bound *O*-acyltransferase domain-containing 7 (MBOAT7) and the glucokinase regulator (GCKR) gene locus^28^. Thus, it is possible that breeder-associated heterogeneity in NASH development can be partly explained by gene variants and epigenetic profile, but this remains to be investigated in guinea pigs.

The relative weight-gain in animals from the two vendors was similar at week 16 and, though not reaching statistical significance appear, nominally higher in animals from Envigo at week 24. This separates the severity of the hepatic lesions from adiposity in this model, which is in agreement with NASH also being present in lean individuals^29,30^. In *foz/foz* mice, the C57BL/6 mouse strain developed more severe NASH and fibrosis compared with a BALB/c strain, and also had hypercholesterolemia and insulin resistance^31^. In mice, genes associated with low-density lipoprotein cholesterol increased in parallel with genes associated with hepatic fibrosis^4^. Thus, a more perturbed metabolic profile may facilitate NASH progression^31^. Accordingly, while guinea pigs from both breeders exhibited hypercholesterolemia, circulating cholesterol levels were higher in animals from Charles River and could help explain the more severe liver phenotype in these animals. Breeder differences in levels of AST, ALP and plasma cholesterol were also observed in chow fed guinea pigs, but these ‘baseline differences’ was not the underlying cause of the differences between breeders observed in animals with NASH. Highlighting that differences can be present even in healthy animals further emphasize the discordances between vendors and that researchers should be conscientious of the source of their research animals.

As a retrospective investigation, using previously collected data from independent studies pose a risk of introducing bias. For the current work, all studies were conducted at the same housing facilities, with identical diets from the same manufacturer and by the same animal caretakers adhering to standard operating procedures from our research group. Additionally, the included studies were dispersed throughout the duration of the entire period independent of breeder, hence not clustered—e.g. with studies using animals from one breeder at the beginning/end-making it unlikely that the observed differences are caused by time-dependent factors. All sample collection, preparation and analyses were performed in a randomized order and with animal identification and group allocation undisclosed during analyses (blinding). Furthermore, liver histology from all studies was scored by the same observer to eliminate inter-observer differences. When analysing the included end-points, the individual animals did not group according to study (Supplemental Figures S1–S3—except for hepatic cholesterol levels in Envigo animals at week 24/25), supporting that the present findings are not caused by a single study with extreme values. Together, this supports the integrity of our findings and that the recorded differences are not due to experimental differences or flaws leading to biased interpretations. Due to the retrospective nature of the current study, we are unable to infer causality but rather underline a potentially important source of irreproducibility in preclincal research. Future prospective studies should seek to determine the underlying reasons for the observed differences, e.g. by investigating the impact of the gut microbiota, adipose tissue dysfunction, circulating adipokines such as leptin, and genetic variation.

In conclusion, our data convincingly demonstrate breeder-specific impact on NASH development in commercially available guinea pigs from two different breeders. Thus, care should be taken when uncritically comparing findings between studies applying different sources of animals. Differences in breeders could be a significant contributor to increased variation and reduced reproducibility in preclinical NASH models, including the guinea pig, and should be taken into account when choosing an experimental model.

## Methods

All animal experimentations were approved by the Animal Experimentation Inspectorate under the Danish Ministry of Environment and Food, and in accordance with the European Legislation of Animal Experimentation 2010/63/EU and carried out in compliance with the ARRIVE guidelines.

### Animals and experimental design

**Figure 6:**
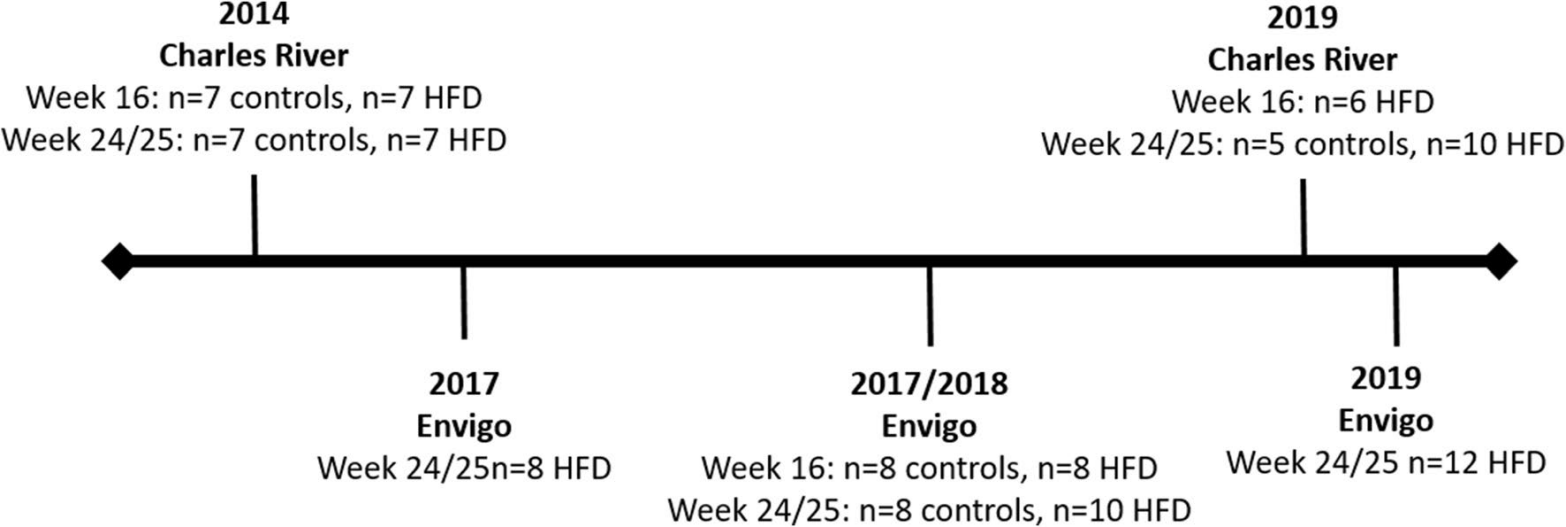
Overview of included studies. This retrospective analysis included animals from five different studies conducted from 2014 to 2019. Studies were not executed sequentially e.g. with animals from one breeder at the start and animals from the other breeder at the end of the time period, minimizing the risk of bias.

This retrospective study is based on previously published data^12,14^ and unpublished data from current studies conducted from 2014 to 2019 and compares Hartley/Dunkin–Hartley female guinea pigs from the two major commercial breeders in the EU: Charles River Laboratories (Lyon, France) and Envigo (Horst, The Netherlands) (Fig. 6). All studies were conducted by the same personal at the same facilities with identical housing condition, using feed from only one manufacturer (Ssniff Spezialdiäten GmbH, Soest, Germany). For all studies, animals were block randomized based on weight into different groups and housed in floor pens with wood shaving, hay, water, gnawing blocks and shelters. Animals were maintained on a 12 h light–dark cycle with temperatures between 20 and 24 °C. Guinea pigs were 10–12 weeks of age when fed a high fat diet (20% fat, 15% sucrose and 0.35% cholesterol) for 16 or 24/25 weeks. At week 16, the study encompasses 13 and 8 high fat fed animals from Charles River and Envigo, respectively. At week 24/25, 17 and 30 high fat fed animals were included from Charles River and Envigo, respectively. For reference values, parallel chow fed (4% fat, 0% sucrose and 0% cholesterol) controls are included from both breeders at week 16 (n_Charles River_ = 7 and n_Envigo_ = 8) and week 24/25 (n_Charles River_ = 12 and n_Envigo_ = 8). Diets were stored at − 20 °C and food batches freshly thawed twice weekly. The exact dietary compositions have been previously described^12^ After 16 or 24/25 weeks, animals were pre-anaesthetized with 0.8–1.25 ml/kg Zoletil-mix, placed on isoflurane (3–5%) and euthanized by decapitation as previously described^12,14^.

### Plasma and liver samples

Plasma and liver samples were prepared and analysed as described previously^12,14^. Briefly, an intracardial blood sample was collected in a K_3_-EDTA flushed syringe prior to euthanization. Blood samples for alkaline phosphatase (ALP) and free fatty acids were collected in heparin- and NaF-microvettes (Sarstedt, Nümbrecht, Germany), respectively. All liver samples were collected from the middle part of the left lateral lobe (*lobus hepatis sinister lateralis*) and fixed in formaldehyde for histology or frozen and stored at − 80 °C prior to measurement of hepatic triglyceride and cholesterol levels. Plasma was isolated and analysed for total cholesterol, triglycerides, free fatty acids, ALT, AST and ALP and liver homogenates were analysed for total cholesterol and triglycerides using a Cobas 6000 (Roche Diagnostic Systems, Berne, Switzerland) according to the manufactures specifications.

### Liver histology

Liver sections from the left lateral lobe of animals fed the high fat diet were stained with haematoxylin and eosin, Masson’s trichrome or Picro Sirius Red. Six haematoxylin and eosin (two from Envigo and four from Charles River) and five Masson’s trichrome/Picro Sirius Red (two from Envigo and three from Charles River) stained sections were damaged and could not be scored. All slides were re-scored in a randomized and blinded fashion according to Kleiner et al*.*^32^. Afterwards, ten randomly selected slides were scored again and the weighted Cohens Kappa coefficient was calculated to confirm intra-observer agreement. Hepatic steatosis was scored as 0: < 5%, 1: 5–33%, 2: > 33–66% or 3: > 66%. Hepatocellular ballooning was scored as: 0: none, 1: few or 2: many. The number of inflammatory foci (defined as ≥ 3 closely associated inflammatory cells) was scored in five separate lobules (defined by the presence of two portal areas and one central vein) at 20× magnification as 0: none, 1: < 2 foci per lobule, 2: 2–4 foci per lobule or 3: > 4 foci per lobule. Portal inflammation was scored at 20× magnification as 0: < 2 foci per portal area/not present or 1: ≥ 2 foci per portal area/present by evaluating three different portal areas distributed throughout the liver. For portal inflammation, a focus was defined as ≥ 5 inflammatory cells in close association. Fibrosis was scored as 0: none, 1: perisinusoidal or periportal, 2: perisinusoidal and periportal, 3: bridging fibrosis or 4: cirrhosis. The non-alcoholic fatty liver disease (NAFLD) activity score (NAS) was calculated as the unweighted sum of steatosis, ballooning and inflammation. The SAF activity score was calculated as the sum of inflammation and ballooning to dissociate these two lesions, with distinct prognostic information, from steatosis^33^. For calculation of the SAF activity, inflammation was scored as 0: none, 1: < 2 foci per lobule or 2: > 2 foci per lobule and ballooning was scored as described above^33^. Animals were diagnosed with NASH only if they simultaneously displayed steatosis, hepatocellular ballooning and inflammation^33^. Otherwise, they were categorized as non-NASH.

### qPCR

qPCR data was available from a subset of the published^12,34^ and unpublished studies included in this retrospective analysis. Gene expression analysis included control (n_Charles River_ = 7 and n_Envigo_ = 8) and NASH animals (n_Charles River_ = 7 and n_Envigo_ = 8) at week 16 as well as control (n_Charles River_ = 12 and n_Envigo_ = 8) and NASH animals (n_Charles River_ = 17 and n_Envigo_ = 10) at week 24/25. Samples had been run in triplicates using the StepOnePlus Real-Time PCR system (Applied Biosystems, Foster City, CA, USA) using identical conditions and primers as previously described^34^. Previously obtained Ct-values of the included genes (*Ccl2, Cxcl8, Tnf, Pdgfb, Serpine1, Col1a1, Acta2, Eln* and *Hprt*) from different studies were pooled according to group (control or NASH), breeder (Charles River or Envigo) and time (week 16 or 24/25) and then analysed. *Serpine1, Acta2* and *Eln* were not analysed in all studies at week 24/25, thus, n_Charles River, control_ = 7 and n_Charles River, NASH_ = 7 for these genes at this time point. Due to technical difficulties n_Charles River, control_ = 6 for *Acta2*, n_Charles River, NASH_ = 5 for *Cxcl8* and n_Envigo, control_ = 7 for *Serpine1* at week 16 while n_Charles River, control_ = 10 and n_Charles River, control_ = 16 for *Cxcl8* at week 24/25.

### Statistical analyses

All statistical analyses were performed in GraphPad Prism version 8.3.0 for Windows (GraphPad Software, San Diego, California USA). Body weights were analysed by a mixed effects model. Plasma and liver data were analysed by a two-way ANOVA with Sidak’s multiple comparison test. Data are presented as means with standard deviations. In case of inhomogeneous variance, the analyses were performed on log_10_-transformed data, which were subsequently back-transformed and presented as geometric means with 95% confidence intervals. Main effects from the two-way ANOVA were only interpreted if the interaction-term was not statistically significant or a significant interaction was ordinal. If variance homogeneity could not be achieved by transformation, data were analysed using the Mann–Whitney test to compare the two breeders at either week 16 or week 24/25 (only done for liver cholesterol). Ordinal data (liver histology) were analyzed with the Mann–Whitney test. Frequency data was analysed by Fishers exact test at each time-point. Gene expression data were analysed using the ΔΔCt-method and is presented as mean fold changes with ranges on a log_2_-scale^35^. ΔΔCt-values were calculated for animals with NASH relative to respective breeder controls and statistical analysis performed on ΔΔCt-values and their standard deviation using an unpaired t-test or Welch’s t test in case of heterogeneous variance.

## Supplementary Information

Supplementary Information
