# Supplementary material for: The development of nonalcoholic steatohepatitis is subjected to breeder dependent variation in guinea pigs
Source: Sci Rep. 2021 Feb 3;11:2955. doi: 10.1038/s41598-021-82643-0 (PMC7859397; doi:10.1038/s41598-021-82643-0)
Supplement: Supplementary file 1 — Supplementary Information [file 41598_2021_82643_MOESM1_ESM.pdf]

The development of nonalcoholic steatohepatitis is subjected to breeder dependent variation in guinea pigs

Ipsen DH[†], Agerskov RH[†], Klaebel JH, Lykkesfeldt J and Tveden-Nyborg P

[†]These authors contributed equally to the work

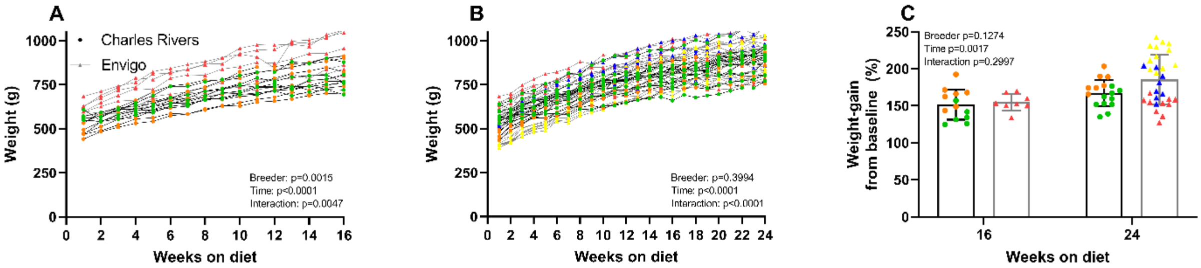

Supplemental Figure S1– Body weight and relative weight-gain with individual studies highlighted

Corresponds to Figure 1. Individual data points from each of the included studies are highlighted. Charles River 2014: Orange, Charles River 2019: Green, Envigo 2017: Blue, Envigo 2017/2018: Red, Envigo 2019: Yellow. Bodyweights from week 1 to 16 (A) and from week 1 to 24 weeks (B). Weight-gain relative to baseline (to account for dissimilar starting weights) (C). No single study contained extreme observations that could bias the results, although at week 24/25 animals from Envigo 2019 (yellow) had a higher weight-gain, these animals were also smaller to begin with. Analysed by mixed effect model (A and B) or 2-way ANOVA (C).

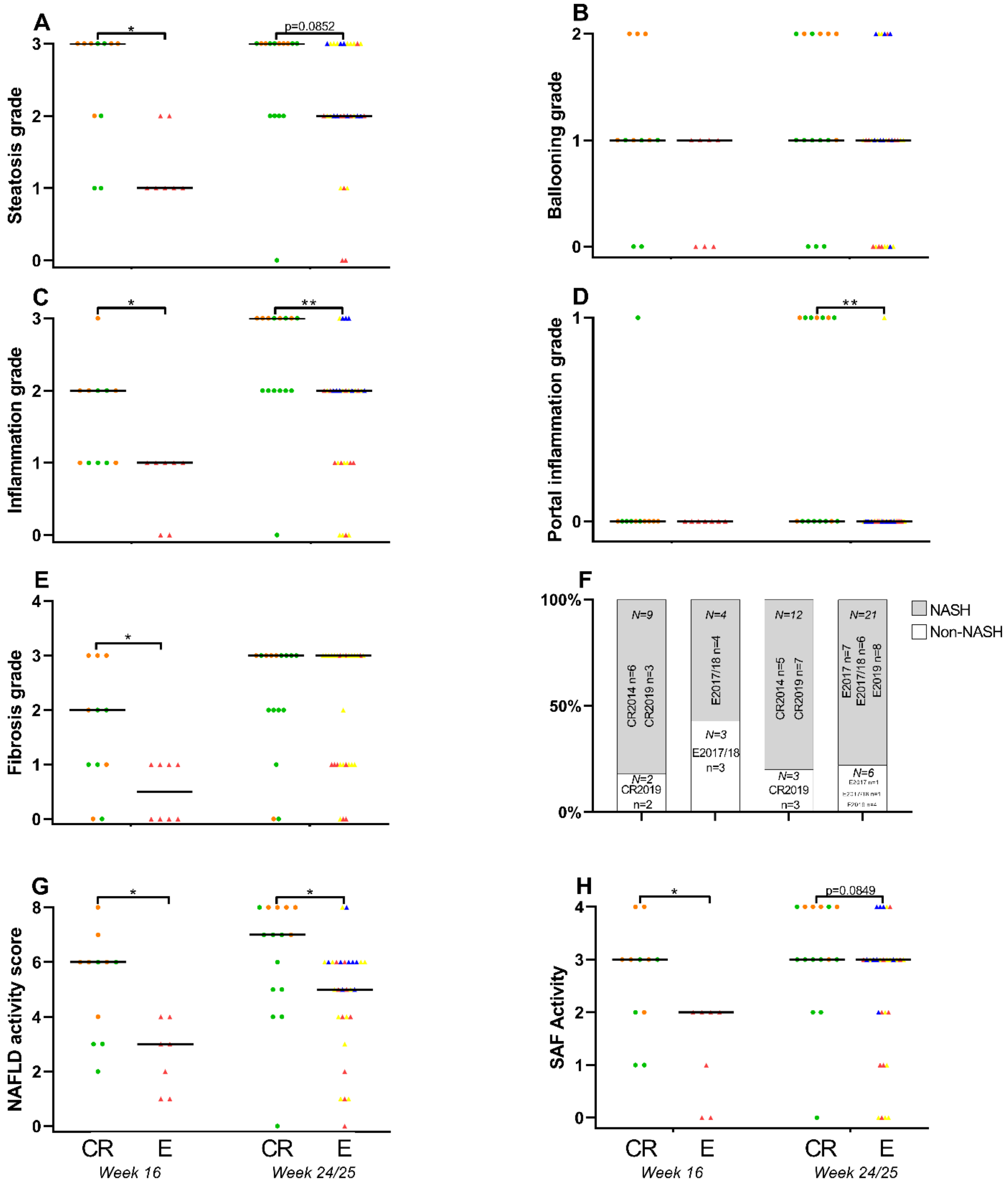

Supplemental Figure S2 – Liver histology and disease severity with individual studies highlighted

Corresponds to Figure 2. Individual data points from each of the included studies are highlighted. Charles River 2014: Orange, Charles River 2019: Green, Envigo 2017: Blue, Envigo 2017/2018: Red, Envigo 2019:

Yellow. No single study contributed with extreme values that could bias the results. *p<0.05 **p<0.01 compared between breeders at the same time-point. Histological scores were analysed at each time-point by Mann-Whitney test (A-E, G and H) and frequencies (F) by Fishers exact test. *CR: Charles River. E: Envigo*.

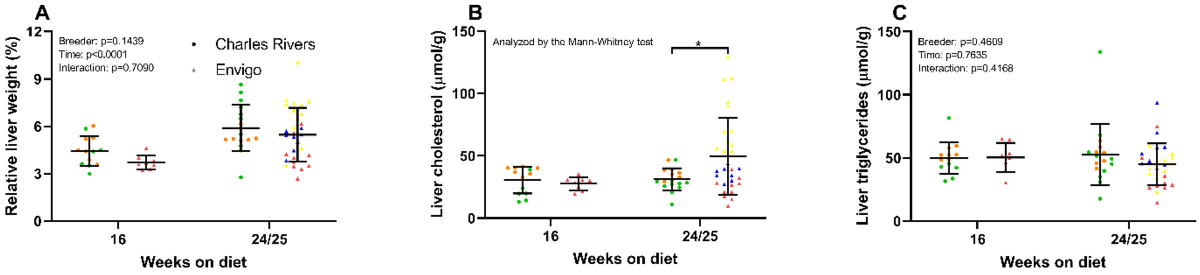

Supplemental Figure S3 – Liver weight and lipid content with individual studies highlighted

Corresponds to Figure 4. Individual data points from each of the included studies are highlighted. Charles River 2014: Orange, Charles River 2019: Green, Envigo 2017: Blue, Envigo 2017/2018: Red, Envigo 2019: Yellow. (A) Liver weight relative to bodyweight was not affected by any single study. (B) Hepatic cholesterol content was higher in Envigo animals compared with Charles River at week 25, but this difference seems to be mainly due to a single study (Envigo 2019, see Methods and Figure 6). (C) Liver triglyceride levels was not impacted by any single study. *$p<0.05$ compared between breeders at the same time-point. Analysed by 2-way ANOVA (A and C) or Mann-Whitney test (B) as variance homogeneity could not be obtained following transformation.
